# Hair Toxic Trace Elements of Residents across the Caspian Oil and Gas Region of Kazakhstan: Cross-Sectional Study

**DOI:** 10.3390/ijerph191811158

**Published:** 2022-09-06

**Authors:** Gulnara Batyrova, Zhenisgul Tlegenova, Victoria Kononets, Gulmira Umarova, Khatimya Kudabayeva, Yerlan Bazargaliyev, Ainur Amanzholkyzy, Yeskendir Umarov

**Affiliations:** 1 Department of Laboratory and Visual Diagnostics, West Kazakhstan Marat Ospanov Medical University, 68 Maresyev Street, Aktobe 030019, Kazakhstan; 2 Department of Internal Diseases No. 2, West Kazakhstan Marat Ospanov Medical University, 68 Maresyev Street, Aktobe 030019, Kazakhstan; 3 Department of Natural Sciences, West Kazakhstan Marat Ospanov Medical University, 68 Maresyev Street, Aktobe 030019, Kazakhstan; 4 Department of Evidence-Based Medicine and Scientific Management, West Kazakhstan Marat Ospanov Medical University, 68 Maresyev Street, Aktobe 030019, Kazakhstan; 5 Department of Internal Diseases No. 1, West Kazakhstan Marat Ospanov Medical University, 68 Maresyev Street, Aktobe 030019, Kazakhstan; 6 Department of Normal Physiology, West Kazakhstan Marat Ospanov Medical University, 68 Maresyev Street, Aktobe 030019, Kazakhstan

**Keywords:** Caspian region, hair analysis, mass spectrometry, oil production, toxic trace elements, western Kazakhstan

## Abstract

This study aimed to assess the relationship between the content of toxic trace elements, such as aluminum (Al), arsenic (As), beryllium (Be), cadmium (Cd), mercury (Hg), and lead (Pb), in the hair of the adult population of western Kazakhstan and the distance of their residence from oil and gas fields. The cross-sectional study included 850 adults aged 18–60 years. Inductively coupled plasma mass spectrometry was used to measure the level of Al, As, Be, Cd, Hg, and Pb in hair. The relationship between the concentration of toxic trace elements in the hair and the distance from oil and gas fields was assessed in three groups (<16 km, 16–110 km, and >110 km), using multiple linear regression analysis. The highest concentration of Hg = 0.338 μg/g was determined in the group living near oil and gas fields (0–16 km), whereas the lowest concentration of Al = 3.127 μg/g and As = 0.028 μg/g was determined in participants living at a long distance (more than 110 km) (*p* < 0.001). The concentration of Al (−0.126 (CI: −0.174; −0.077)), Hg (−0.065 (CI: −0.129; −0.001)), and Pb (0.111 (CI: 0.045; 0.177)) is associated with the distance to oil and gas fields. The obtained data indicate a change in the toxic trace element content in the hair of residents in the Caspian region of western Kazakhstan, a change that is most pronounced in residents living in the zone of oil and gas pollution. The distance to the oil and gas fields affects the content of toxic elements in scalp hair. In particular, the concentration of Al and Hg is associated with a decrease in the distance to oil and gas fields, while the concentration of Pb is associated with an increase in the distance to these fields. The lowest content of Al and As was determined in the hair of study participants living in the most remote areas (more than 110 km from oil and gas fields). Our results demonstrate the need for the biomonitoring of toxic elements to determine long-term temporal trends in the impact of chemicals on public health in western Kazakhstan.

## 1. Introduction

Environmental pollution has increased significantly in recent decades due to the active development of mining, the processing industry, and road transport [1]. A significant source of environmental pollution is the oil industry, which is associated with oil-field exploration, and the production, transportation, storage, and processing of oil and gas [2]. Environmental monitoring studies have confirmed the existence of a health risk not only for workers directly involved in hazardous production, but also for the population as a whole [3].

At all stages of oil and gas production and processing, toxic trace elements are released into the environment, polluting the soil, water, and air [4]. Existing oil fields potentially have an impact on the health and environment of more than 600 million people around the world [5]. The workers involved in cleaning up oil spills and residents of oil and gas fields have described toxic effects in the form of headache, intoxication, and respiratory, skin, and eye symptoms [6]. Excessive exposure to oil products leads to cardiovascular [7] and respiratory diseases [8], pathology of the nervous system [9], and endocrine and genetic damage [10].

Oil production is an important source of environmental pollution with elemental Hg [11,12], Pb [13,14,15], As [16,17], Cd [14], Be [18,19], and Al [20,21,22]. The most studied toxic trace elements are Pb, Cd, Hg, and As. When entering the human body, even in small amounts, these trace elements have a systemic toxic effect [23,24].

The content of heavy metals in the blood is used as a biomarker in assessing the potential risk of exposure to oil [25]. However, body fluids, such as urine, blood, saliva, and lacrimal fluid, reflect only its short-term state, that is, the state at exactly the point of measurement. Unlike liquid biomarkers of exposure, the deposition of trace elements in the hair provides more accurate determination [11]. According to a study in Punjab, the results of the analysis of trace elements in hair and urine confirm their correlation with each other [26]. Blaurock-Busch et al. believe that if the immediate exposure, as determined by the level of toxic trace elements in the urine, persists for weeks, months, or longer periods, it contributes to and actually causes long-term exposure, which reflects the ability of the hair to act as a biosubstrate for determining the level of toxic metals in the body [27]. The concentration of toxic trace elements in the hair reflects the characteristics of the environment, including long-term exposure to adverse environmental factors [28]. In a number of studies, hair is used as a biosubstrate for the determination of toxic metals in the human body [11,13,29,30,31,32].

The oil sector of the Republic of Kazakhstan accounts for 25% of the gross domestic product and the country is eighth in the world in terms of oil reserves. The intensive development of oil and gas fields has led to the deterioration of the environment. Kazakhstan ranks 84th in the Environmental Performance Index and 137th in the Life Expectancy Index [33]. The largest oil and gas fields in Kazakhstan are concentrated in the west of the country and are often located near settlements. The inhabitants of these settlements have been exposed to the adverse effects of oil and gas for a long time.

There are no well-equipped landfills for the disposal of waste from the oil industry on the studied territory of western Kazakhstan. Some oil-producing companies are discharging formation water to evaporation fields without treatment, and some companies have changed their method of disposal to underground injection into abandoned wells. The northern region of the Caspian Sea has repeatedly become the site of oil spills at various stages of oil production—at the stage of drilling wells and oil extraction and the stage of the conservation of exhausted wells—which has led to significant pollution of the adjacent territories by oil products. In the areas where petrochemical complexes are located, higher concentrations of Cd and Pb in the soil are described [34], wastewater is significantly enriched in Be and Li [18], and formation waters contain high concentrations of Cd and Pb [35]. In this regard, there is a need to study the level of toxic trace elements in the biosubstrates of the population of the oil and gas production region in western Kazakhstan.

This study aimed to explore the content of the toxic metals, namely aluminum, arsenic, beryllium, cadmium, mercury, and lead, in the hair of residents of western Kazakhstan, depending on the remoteness of their location from oil and gas fields.

## 2. Materials and Methods

### 2.1. Study Area and Participants

**Figure 1:**
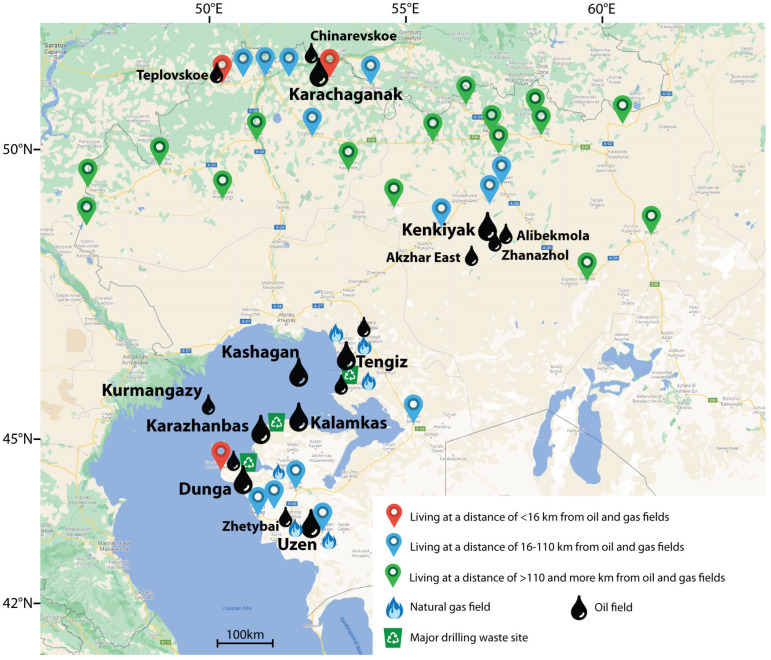
Map of the surveyed areas [36].

A cross-sectional study using a cluster sample was carried out in Aktobe, Mangistau, and western Kazakhstan provinces (oblasts) of the Republic of Kazakhstan. The study included 850 permanent residents aged 18–60 years. Detailed description of the settlements and recruitment procedures have been presented in our earlier publications [36]. Depending on the distance of residence from oil and gas fields, a comparison was made of the content of toxic elements Al, As, Be, Hg, Cd, and Pb in three groups: less than 16 km, from 16–110 km, and over 110 km. The activities of an oil and gas enterprise affect the human body at a distance of up to 16 km [37], and settlements located more than 110 km away were considered relatively unpolluted [13]. The distance from settlements to deposits was determined using Google Maps. For each settlement, the coordinates of the centers were taken from Wikipedia (Figure 1).

Informed consent was obtained from each participant. The study was approved by the local ethics committee of the Marat Ospanov West Kazakhstan Medical University (meeting No. 5 dated 13 May 2020) and performed according to the principles of the Helsinki Declaration and subsequent amendments.

### 2.2. Hair Samples and Analysis

Hair collection was carried out between November 2020 and February 2021. Occipital hair samples of at least 0.1 g were obtained by cutting with clean stainless steel scissors. Only proximal parts of the collected hair strands (1–2 cm) were used for analysis. Hair samples were stored in a paper envelope until analysis.

In the laboratory, hair samples were subject to preparation by washing and microwave decomposition. The hair strands were washed with acetone, then rinsed three times with deionized water (18 MΩ cm) from a DVS-M/1HA-1(2)-L electric distiller (Mediana-Filter, Podolsk, Russia). The use of acetone as a washing agent removes dirt and dust from hair samples without influencing endogenously bound trace elements, as shown in earlier studies [38,39]. Washed hair samples were dried on air at 60 °C to a stable weight. A total of 50 mg dry washed hair samples were introduced into nitric-acid-precleaned Teflon containers containing 5 mL of concentrated (65%) nitric acid (Sigma-Aldrich Co., St. Louis, MO, USA). Microwave digestion was carried out for 20 min at a temperature of 170–180 °C in a “Berghof Speedwave 4 system” (Berghof Products + Instruments, GmbH, 72800 Eningen, Germany). After cooling and equalizing the pressure in the system, the solutions obtained during decomposition were transferred into tubes, and the volume was adjusted to 15 mL with distilled deionized water (18 MΩ cm). The final solution was used for chemical analysis.

The concentration of toxic chemical elements, Al, As, Be, Hg, Cd, and Pb, was determined by inductively coupled plasma mass spectrometry (ICP MS) using a NexION 300D mass spectrometer (PerkinElmer Inc., Shelton, CT, USA) equipped with 7-port FASTvalve and ESI SC-2 DX4 autosampler (Elemental Scientific Inc., Omaha, NE, USA). The ICP-MS system was conditioned and calibrated in accordance with the manufacturer’s manual via external calibration. The external calibration solutions containing 0.5, 5, 10, and 50 μg/L of the studied elements were freshly prepared from the Universal Data Acquisition Standards Kits (PerkinElmer Inc., Shelton, CT, USA) by dilution with distilled deionized water and subsequent acidification with 1% HNO_3_ (Sigma-Aldrich Co., St. Louis, MO, USA). Internal online standardization using 10 μg/L solutions of yttrium-89 and rhodium-103 was performed. The solutions were prepared from Yttrium (Y) and Rhodium (Rh) Pure Single-Element Standard (PerkinElmer Inc. Shelton, CT, USA) on a matrix containing 8% 1-butanol (Merck KGaA, Gernsheim, Germany), 0.8% Triton X-100 (Sigma-Aldrich Co., St. Louis, MO, USA), 0.02% tetramethylammonium hydroxide (Alfa Aesar, Ward Hill, MA, USA), and 0.02% ethylenediaminetetraacetic acid (Sigma-Aldrich Co., St. Louis, MO, USA).

Certified reference material of human hair GBW09101 (Shanghai Institute of Nuclear Research, Shanghai, China) was used for laboratory quality control (Table 1). The recovery rates for all studied toxic chemical elements were within the range of 90–110%.

### 2.3. Statistical Analysis

The distributions of the data concentrations of the toxic trace elements (Al, As, Be, Cd, Hg, Pb) in hair samples was non-Gaussian. The content of toxic trace elements in hair is presented with the geometric means (GM), arithmetic means (AM), median (Me), percentiles (P2.5%; P97.5%), maximum (Max), and minimum (Min) values. We analyzed the differences in the content of toxic trace elements in three groups depending on the place of residence (<16 km, 16–110 km and >110 km) using the Kruskal–Wallis H-test. For a posteriori comparisons for these 3 groups, a new critical level *p* < 0.017 was used [40].

Linear regression analysis was used to assess the relationship between concentrations of toxic trace elements in the hair and the settlement’s remoteness from the oil and gas production site (model 0). To perform linear regression analysis, we transformed the data using the natural logarithm Ln(X). In the multiple linear regression analysis, important factors, such as age, gender, body mass index (BMI), and tobacco smoking (model A,B,C), were included. For testing statistical hypotheses, the critical significance level, *p*, was taken as equal to 0.05. SPPS.v.25 Modeler (IBM) and Statistica.v.10 (StatSoft, Tulsa, OK, USA) software were used for statistical analysis.

## 3. Results

### 3.1. Toxic Trace Element Content in Hair of Residents of Western Kazakhstan

The study included *n* = 850 representatives of the adult population from 32 settlements in western Kazakhstan. The distance of settlements from the oil and gas production area ranged from 2.3 to 475 km. The average age of the participants was 42 (31.0; 53.0) years. Among participants were 350 (41.2%) men, and 239 (28.1%) of the surveyed population lived in the city [36].

Table 2 shows the concentration of Al, As, Be, Cd, Hg, and Pb in the hair of residents of western Kazakhstan. Data are presented in three groups depending on the remoteness of oil and gas fields from the place of permanent residence of the participants. There were significant differences in the content of Al, As, and Hg in the hair between the three groups. The lowest concentration of Al and As was found in the hair of study participants from the most remote settlements (more than 110 km from oil and gas fields). The content of Hg in the hair of residents living near oil and gas fields (0–16 km) was 2.5 times higher than that of residents from the settlements furthest from the fields (more than 110 km). The concentration of Be, Cd, and Pb in the hair of representatives of the three studied groups living at different distances from oil and gas fields showed no significant differences.

### 3.2. Effects of the Remoteness of the Oil and Gas Production/Processing Sites on Levels of Toxic Trace Elements in Hair

Using multiple linear regression analysis, we examined how the relationship between toxic trace element concentrations in hair and distance changes when the effect of variables, such as age, gender, BMI, and smoking, is taken into account. The results are presented in Table 3. To demonstrate how this table should be understood, we use the example of Pb. In Model 0, the regression coefficient of Pb, 0.084 (95% CI 0.010; 0.159), means that the natural logarithm of the concentration of Pb in the hair of residents of western Kazakhstan increases by the indicated value with each additional 100 km. A distance of 100 km was chosen in all models to obtain more significant linear regression coefficients. Adjusted regression coefficients represent the change in hair Pb concentration due to distance after age and sex were taken into account in Model A; age, sex, and BMI in Model B; and age, sex, BMI, and smoking in Model C (Table 3).

Using multiple linear regression analysis, distance was found to be significantly associated with hair concentrations of Al and Hg (−0.126 (95% CI: −0.174; −0.077) and −0.065 (95% CI: −0.129; −0.001), respectively). The regression coefficients indicated a lower concentration of these trace elements in the hair of residents living at a greater distance from oil and gas production and processing sites.

## 4. Discussion

We determined the content of toxic trace elements such as Al, As, Be, Cd, Hg, and Pb in the hair of residents of the Caspian region in Kazakhstan, living at different distances from oil and gas production fields (0–16 km, 16–110 km, and more than 110 km). The examined groups had the greatest differences in the content of Al, As, and Hg in their hair, wherein the lowest concentration of these trace elements was in the study participants living at a great distance (more than 110 km) from oil and gas field settlements (Table 2).

Among the toxic trace elements, Al is rarely the focus of attention during the biomonitoring of human populations in regions of oil and gas pollution. Moon et al. indicated that no difference was found in the content of Al in the hair of children from a region close to an oil field and their peers from control areas [41]. On the contrary, Saleh et al. claimed that there is an increased level of Al in hair and blood [42] in residents of regions of oil and gas production, which is similar to the results from this study. Moreover, Caron-Beaudoin et al. report a significant (three-fold) increase in the Al concentration in the hair of pregnant women living in an oil production area compared with the control group [20].

Gonzalez et al. and Yuan et al. found that residents of oil provinces have a higher concentration of As not only in hair, but also in other biosubstrates [16,17]. The authors, as is also the case in this study, established a tendency for the As concentration in biosubstrates, including the hair of residents, to change depending on the distance to oil and gas fields [16,17]. However, an increase in the As level in the hair of residents was noted not only in oil- and gas-producing regions, but also in agricultural regions [11]. At the same time, Skalny et al. obtained opposing results by comparing the As content in the hair samples of petrochemical plant workers (0.033 µg/g) and a control group living at a distance of 16 km from the plant (0.053 µg/g), as well as office workers (0.062 µg/g) [43].

According to Relic et al., one of the hazardous soil pollutants in areas close to the petrochemical industry is Hg [14]. O’Callaghan-Gordo et al. observed high concentrations of Hg in the urine of residents living in areas where oil spills occurred [12]. Webb et al. showed that indigenous Peruvians and Ecuadorian Amazonians from oil extraction sites often had urine Hg levels within the World Health Organization’s global background standard. However, the level of Hg in the urine was increased in men who worked in the liquidation of oil spills and in women who used surface water for household use [44]. In addition, a significant increase in the concentration of Hg in the blood of residents of the oil and gas region is also reported by Saleh et al. [42]. Similarly, this study found a significant increase in Hg concentration in the hair of residents living near oil and gas fields (0–16 km), exceeding the same indicator in residents of areas far from the fields by more than 2.5 times (Table 2). According to some researchers, hair is a suitable biomarker specifically for assessing the concentration of toxic trace elements in the body [45].

In our study, there were no differences in the concentrations of Be, Cd, and Pb in the hair of examined residents living in territories of varying degrees of remoteness from oil and gas fields (Table 2). This is only partially consistent with the results of similar studies conducted in populations living in areas contaminated by oil and gas products. An increase in the concentration of Cd in the biosubstrates of residents of territories contaminated with oil products after oil spills was reported by O’Callaghan-Gordo et al. [12]. The difference in the obtained results may be due to the use of different biosubstrates for analysis. In a study by O’Callaghan-Gordo et al., urine was used as a biosubstrate for the analysis of trace element contents. Levels of toxic trace elements in hair do not correlate with levels found in urine [12]. Hair deposits of trace elements reflect quantitative changes in trace elements and related metabolic processes over a long period, whereas the content of trace elements in urine, saliva, lacrimal fluid, and blood reflects the short-term trace element status of the organism. Hair samples are widely used to assess human exposure to various pollutants due to their many advantages. A number of authors have questioned the reliability of this biomarker for assessing the level of trace elements in the human body. For example, Rodrigues et al. do not consider hair to be an appropriate biomarker to assess Cu, Mn, and Sr deficiency or Pb exposure [29]. The data obtained in our study to determine the concentration of Pb in the hair of the population are similar to the results of the study by Anticona et al., who reported no increase in the level of Pb in the biosubstrates of an Amazonian population living in a region affected by the oil industry [46].

Changes in the concentration of Be in the biosubstrates of residents of oil and gas production regions remain poorly understood. According to Vethanayagam et al., working in the oil industry is associated with the risk of intoxication with Be [47]. A study performed by Skalny et al. showed that the level of Be in the hair of employees of a petrochemical plant was significantly lower than in the control group, who did not work in oil refining [43]. Our study did not establish a difference in the accumulation of Be in the hair of residents of regions with different degrees of remoteness from oil and gas fields (Table 2).

The results of a comparative analysis of the content of toxic trace elements Al, As, Be, Cd, Hg, and Pb in the hair of residents of western Kazakhstan living in three zones with varying degrees of remoteness from oil and gas production and processing sources were confirmed by multiple linear regression analysis. In this analysis, with increasing distance from the place of residence to oil and gas fields, the concentrations of Al, As, and Hg in the hair of the study participants decreased. The models developed for Pb describe the opposite relationship, indicating an increase in the concentration of Pb in the hair with distance from the alleged source of contamination. For As, only the unadjusted model (Model 0) was significant, in which the distance from the place of residence to the point of oil and gas production was used as a predictor variable (Table 3). In constructing Models A, B, and C, age, gender, BMI, and smoking were introduced as confounders. Perhaps the distribution of As in hair associated with the influence of these factors can explain the statistical significance of Model 0 for As, and its disappearance when the above corrections are introduced. The concentration of toxic trace elements in hair depends not only on their intake with food and from the environment, but also on many additional endogenous and exogenous factors.

Table 4 shows the results of determining the concentration of the toxic trace elements Al, As, Be, Cd, Hg, and Pb in the hair of residents of different countries. These data indicate the existence of a significant difference between the populations in the distribution of toxic trace elements. This may be due not only to differences in the level and characteristics of anthropogenic factors (industrial, agricultural, and urban pollution) acting in these territories, but also to the natural intake of toxic trace elements from the environment. A decrease or increase in the concentration of toxic trace elements in the body may be associated with living in biogeochemical provinces characterized by an excess or deficiency of certain trace elements. Volcanic activity, the weathering of rocks, geothermal waters, and forest fires are some of the natural sources of toxic trace elements entering the human body [23].

**Figure 2:**
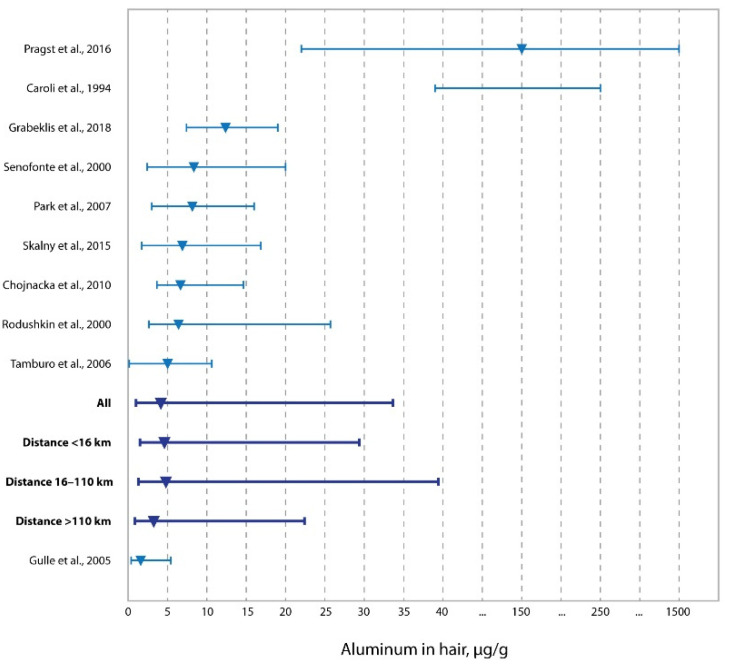
Concentrations of aluminum in the hair of residents living at different distances from oil and gas production fields vs. literature data (medians and ranges) [13,48,49,50,52,54,55,56,57,58].

In Figure 2, the distribution of concentrations of Al in the hair of the adult population of western Kazakhstan living in areas of different distances from oil and gas production and processing enterprises is shown, in comparison with similar data on children of the same region [49], and populations of Italy [58], Russia [48], Sweden [56], Poland [57], Canada [52], South Korea [55], and South Sudan [13], as well as the study by Caroli et al., which consolidated the results of several studies [50]. In the presented populations, children from Italy living in the region of industrial pollution and adults of South Sudan should be considered exposed populations, while the rest of the populations are not exposed or include both exposed and unexposed populations (Caroli et al.) [50]. Noteworthy is the lower median Al content in our study (4.08 μg/g) compared with the rest of the populations, with the exception of the study by Gulle et al. (1.63 µg/g). The increase in the level of Al in the hair of the adult population of western Kazakhstan is expressed both in the zone of oil and gas production (0–16 km) and in the more remote zone (16–110 km). Despite the low median of the concentration of Al in the hair of the population of western Kazakhstan, the upper limit of the ranks is significantly higher than in other studies, with the exception of studies by Pragst et al. and Caroli et al. [13,50]. The study by Pragst et al. was conducted in an extremely oil-polluted region of South Sudan [13], and the study by Caroli et al. consolidated a number of studies, including results from both uncontaminated and polluted regions [50].

**Figure 3:**
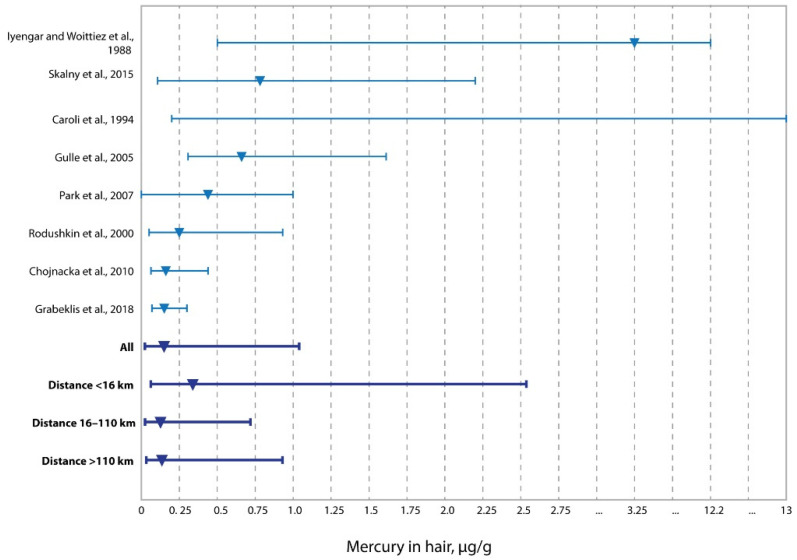
Concentrations of mercury in the hair of residents living at different distances from oil and gas production fields vs. literature data (medians and ranges) [48,49,50,51,52,55,56,57].

The median distribution of Hg concentration in the hair of the adults of western Kazakhstan does not differ from that previously described in children of the same region [49]. However, it is significantly lower than that of a similar indicator in the populations of Russia [48], Sweden [56], Poland [57], South Korea [55], and Canada [52], and data presented by Iyengar and Woittiez, 1988 [51] (Figure 3). The difference in the level of Hg in the hair of residents between the zones of western Kazakhstan, which differ in distance from oil and gas production enterprises, is very significant. In the zone of oil and gas production (0–16 km), the maximum concentration of Hg in hair in the region of western Kazakhstan was noted (0.338 μg/g), which makes it possible to assume that the change in the concentration of Hg in hair is related to the oil industry.

**Figure 4:**
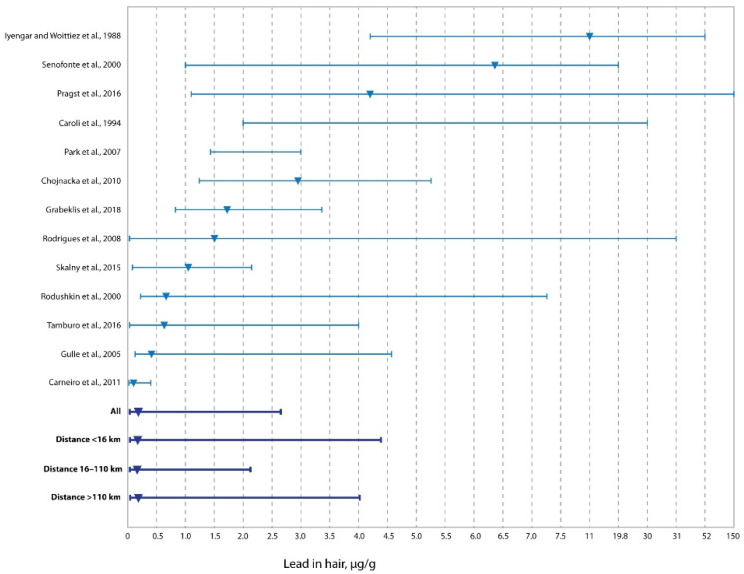
Concentrations of lead in the hair of residents living at different distances from oil and gas production fields vs. literature data (medians and ranges) [13,29,48,49,50,51,52,53,54,55,56,57,58].

The distribution of Pb concentration in the hair of the residents of western Kazakhstan, as shown in Figure 4, demonstrates a pronounced decrease in comparison with similar data of other populations, with the exception of that described for adolescents in Brazil [53]. It should be noted that a relatively low level of Pb in the hair of residents of western Kazakhstan was noted in all the zones identified in this study, including the zone of active oil and gas production (0–16 km). Most of the populations compared in Figure 4 are unexposed, not associated with oil and gas production, which, together with the results of regression analysis for Pb, suggests that the increase in the concentration of Pb in the hair of residents is associated with the action of some other factors and requires further investigation.

This study has some limitations. First, there was a diverse heterogeneity of age and gender in the sample. This is due to men mostly refusing to participate in the study because of having short hair or being bald. On the contrary, women were more willing to consent to participate in the study. Second, complete information about the concentration of the chemical elements in the air, soil, and water of western Kazakhstan was not obtained. Third, we did not take into account dietary habits, which is an important factor as trace element status is also influenced by dietary intake. Finally, residents of the Atyrau oblast, which is also an oil-producing region, were not included in the study. The use of milli-Q quality water and high-purity acid solution would provide better sample clearance. Consequently, further studies are required to investigate the influence of environmental factors and dietary intake on trace elements in hair, with good representation across all age and gender groups. Despite these limitations, this is the first study to have evaluated the correlation between the distance to oil and gas production enterprises and the concentration of toxic trace elements in the hair of people in the Caspian region of western Kazakhstan.

## 5. Conclusions

To conclude, the obtained data indicate a change in the toxic trace element contents in the hair of residents in the Caspian region of western Kazakhstan, a change that is most pronounced in residents living in the zone of oil and gas pollution. The distance to the oil and gas fields affects the content of toxic elements in scalp hair. In particular, the concentration of Al and Hg is associated with a decrease in the distance to oil and gas fields, while the concentration of Pb is associated with an increase in the distance to these fields. The lowest content of Al and As was found in the hair of study participants living in the most remote settlements (more than 110 km from oil and gas fields). Our results demonstrate the need for the biomonitoring of toxic elements to determine long-term temporal trends in the impact of chemicals on public health in the Caspian region of western Kazakhstan.

## Figures and Tables

**Table 1:** Laboratory quality control with certified reference material of human hair GBW09101.

Element	Certified Value, µg/g	Obtained Value, µg/g	Recovery Rate, %	LoD, ppb	BEC, ppb
Al	23.2	23.3 ± 2.48	101	0.13	0.26
As	0.198	0.194 ± 0.014	97	0.001	0.001
Be	n.s.	0.0015 ± 0.0008	-	n.s.	n.s.
Cd	0.072	0.066 ± 0.006	92	0.0016	0.0006
Hg	1.06	1.15 ± 0.29	108	0.012	0.034
Pb	3.83	4.13 ± 0.28	108	0.0006	0.0007

LoD—limit of detection, BEC—background equivalent concentration, ppb—part per billion, n.s.—not specified.

**Table 2:** Comparative analysis of the concentration of toxic trace elements (µg/g) in the hair of residents of western Kazakhstan in 3 groups living at different distances from oil and gas production fields.

Element	Remoteness < 16 km (*n* = 79)				Remoteness 16–110 km (*n* = 422)				Remoteness > 110 km (*n* = 349)				*p* K-W
	AM	GM	Me (Q1; Q3)	P2.5; P97.5	AM	GM	Me (Q1; Q3),	P2.5; P97.5	AM	GM	Me (Q1; Q3),	P2.5; P97.5	
Al	7.158	5.240	4.795 (3.058; 8.601) ^c^	(1.447; 29.345)	8.509	5.373	4.935 (2.905; 8.896) ^c^	(1.214; 39.451)	5.163	3.463	3.127 (1.868; 5.778) ^a,b^	(0.877; 22.346)	<0.001
As	0.034	0.026	0.030 (0.017; 0.047)	(0.004; 0.102)	0.041	0.031	0.034 (0.020; 0.052) ^c^	(0.004; 0.141)	0.033	0.023	0.028 (0.013; 0.043) ^b^	(0.003; 0.097)	<0.001
Be	0.0006	0.0004	0.0004 (0.0001; 0.0008)	(0.0001; 0.0025)	0.0006	0.0004	0.0003 (0.0001; 0.0009)	(0.0001; 0.0027)	0.001	0.0004	0.0004 (0.0001; 0.0009)	(0.0001; 0.0026)	0.605
Cd	0.028	0.012	0.010 (0.005; 0.020)	(0.001; 0.235)	0.026	0.010	0.009 (0.005; 0.019)	(0.001; 0.121)	0.025	0.011	0.011 (0.005; 0.026)	(0.001; 0.127)	0.107
Hg	0.552	0.335	0.338 (0.150; 0.799) ^b,c^	(0.057; 2.529)	0.185	0.121	0.132 (0.062; 0.240) ^a,c*^	(0.017; 0.715)	0.219	0.147	0.139 (0.083; 0.262) ^a,b*^	(0.026; 0.928)	<0.001
Pb	0.574	0.206	0.178 (0.073; 0.417)	(0.028; 4.387)	0.348	0.180	0.161 (0.077; 0.356)	(0.029; 2.126)	0.520	0.217	0.190 (0.099; 0.404)	(0.039; 4.025)	0.140

AM—arithmetic mean; GM—geometric mean. Post hoc comparisons: differences at the level of *p* < 0.001 for ^a,b,^ and ^c^; *p* = 0.013 for ^b*^ and ^c*^; ^a^—<16 km; ^b^—16–110 km; ^c^—>110 km.

**Table 3:** Assessment of crude and adjusted differences in the hair content of toxic trace elements: results of multiple regression analysis.

Element	Model 0	95% CI	*p*	Model A	95% CI	*p*	Model B	95% CI	*p*	Model C	95% CI	*p*
Al	−0.160	−0.216; −0.103	<0.001	−0.127	−0.176; −0.079	<0.000	−0.125	−0.174; −0.077	<0.000	−0.126	−0.174; −0.077	<0.000
As	−0.069	−0.126; −0.012	0.018	−0.024	−0.073; 0.026	0.346	−0.021	−0.071; 0.028	0.397	−0.021	−0.071; 0.028	0.400
Be	0.031	−0.037; 0.099	0.373	0.047	−0.020; 0.114	0.170	0.049	−0.019; 0.116	0.157	0.047	−0.020; 0.115	0.169
Cd	0.040	−0.039; 0.118	0.323	0.059	−0.013; 0.131	0.107	0.063	−0.010; 0.135	0.089	0.064	−0.009; 0.136	0.084
Hg	−0.064	−0.128; 0.000	0.050	−0.076	−0.140; −0.011,	0.021	−0.068	−0.132; −0.004	0.038	−0.065	−0.129; −0.001	0.047
Pb	0.084	0.010; 0.159	0.026	0.107	0.041; 0.172	0.002	0.111	0.046; 0.177	0.001	0.111	0.045; 0.177	0.001

Model 0: Adjusted for distance; Model A: Adjusted for age and gender; Model B: As in Model A, with the addition of BMI; Model C: As in Model B, with the addition of smoking.

**Table 4:** Summary of published data on concentrations of toxic trace elements (μg/g) in hair in different populations.

Sample Type and Location	Al Median (Range)	As Median (Range)	Be Median (Range)	Cd Median (Range)	Hg Median (Range)	Pb Median (Range)	References
Present study, *n* = 850	4.080 (1.007; 33.586)	0.030 (0.004; 0.116)	0.0004 (0.0001; 0.0026)	0.010 (0.001; 0.141)	0.145 (0.019; 1.035)	0.181 (0.032; 2.651)	
Occupationally non-exposed Russian adult population, *n* = 5908	6.936 (1.785; 16.958)	0.045 (0.007; 0.135)	0.003 (0.000; 0.011)	0.034 (0.003; 0.090)	0.775 (0.106; 2.212)	1.046 (0.088; 2.142)	Skalny et al., 2015 [48]
Children aged 7–11 years from Kazakhstan, *n* = 836	12.4 (7.4; 19)	0.081 (0.047; 0.13)	0.0015 (0.0009; 0.0037)	0.063 (0.033; 0.13)	0.145 (0.069; 0.297)	1.72 (0.82; 3.36)	Grabeklis et al., 2018 [49]
Children and adults selected from various countries	(39; 250)	(0.2; 36)	-	(0.7; 4.6)	(0.2; 13)	(2; 30)	Caroli et al., 1994 [50]
Adult population selected from various countries: nearly 100,000 individuals from 55 countries	-	0.26 (0.085; 0.5)	-	1.15 (0.35; 2.43)	3.25 (0.5; 12.2)	11 (4.2; 52)	Iyengar and Woittiez, 1988 [51]
Canada, adults	1.63 (0.26; 5.30)	0.05 (0.03; 0.08)	0.007 (0.003; 0.012)	0.011 (0.004; 0.17)	0.66 (0.31; 1.66)	0.41 (0.13; 4.57)	Gulle et al., 2005 [52]
South Brazil, teenagers 12–18 y.o, *n* = 126	-	0.006 (0.001; 0.02)	-	0.003 (0.000; 0.02)	-	0.1 (0.009; 0.4)	Carneiro et al., 2011 [53]
Italy, schoolchildren, *n* = 412	8.45 (2.4; 20.0)	0.06 (0.14; 0.24)	-	0.14 (0.04; 0.61)	-	6.36 (1.0; 19.8)	Senofonte et al., 2000 [54]
South Korea, children 3–6 y.o, *n* = 655	8.08 (3; 16)	0.11 (0.05–0.20)	-	0.07 (0.01–0.20)	0.43 (0–1)	1.43 (<3)	Park et al., 2007 [55]
Sweden, children + adults, from 1 year old up to 76, *n* = 114	6.4 (2.7; 25.6)	0.067 (0.034; 0.319)	0.0010 (0.0004; 0.0042)	0.034 (0.010; 0.356)	0.249 (0.053; 0.927)	0.660 (0.22; 7.26)	Rodushkin et al., 2000 [56]
Brazil, adult healthy population *n* = 280	-	-	-	-	-	1.5 (0.02–31)	Rodrigues et al., 2008 [29]
Poland, Wroclaw, students aged 20, *n* = 117	6.73 (3.60; 14.69)	0.760 (0.686; 1.025)	-	0.072 (0.058; 0.124)	0.164 (0.063; 0.437)	2.91 (1.24; 5.25)	Chojnacka et al., 2010 [57]
Sicily, children 11–14 years old, *n* = 943	5.0 (0.01; 10.6)	0.03 (0.0003; 0.17)	-	0.01 (0.0003; 0.18)	-	0.63 (0.03; 4.0)	Tamburo et al., 2016 [58]
South Sudan, adults, *n* = 96	150.00 (22; 1500)	-	-	-	-	4.20 (1.1; 150)	Pragst et al., 2016 [13]

## Data Availability

Data are available from the corresponding author upon request.
